# Establishment of a mouse model with all four clinical features of eosinophilic bronchitis

**DOI:** 10.1038/s41598-020-67475-8

**Published:** 2020-06-29

**Authors:** Liyan Chen, Chenhui Li, Min Peng, Jiaxing Xie, Kefang Lai, Nanshan Zhong

**Affiliations:** State Key Laboratory of Respiratory Disease, National Clinical Research Center for Respiratory Disease, Guangzhou Institute of Respiratory Health, The First Affiliated Hospital of Guangzhou Medical University (Guangzhou Medical University), No. 151 Yanjiang Road, Yuexiu District, Guangzhou, 510120 Guangdong China

**Keywords:** Asthma, Experimental models of disease

## Abstract

Eosinophilic bronchitis (EB) is a clinical disease characterized by chronic cough, airway eosinophil infiltration, and responsive to steroid therapy but with the absence of airway hyperreactivity (AHR). This study established an EB mouse model with all the above features. First, 42 mice were divided into 7 groups to investigate the optimal time interval between cough and AHR detections. Afterward, 28 mice were divided into the asthma, EB, normal saline (NS), and dexamethasone (DXM) groups. Mice were challenged using nasal drops of 200 µg ovalbumin (OVA), 10 µg OVA, NS, or intraperitoneal injections of 5 mg/kg of DXM one hour prior to 10 µg OVA challenge. Airway reactivity was measured 6 h after cough was observed. The frequency of coughs in the asthma and EB groups increased significantly compared to mice in the NS group. After DXM administration, frequency of coughs was significantly decreased compared to mice in the asthma and EB groups. Lung resistance in the asthma group was significantly higher compared to mice in the NS, EB, and DXM groups. Obvious airway eosinophilic inflammation in BALF and lung tissues were observed in the asthma and EB groups, while DXM administration could attenuate airway inflammatory infiltration. In summary, we developed a mouse EB model with all four clinical features of EB by the administration of 10 µg OVA nasal drops.

## Introduction

In 1989, Gibson et. al. observed that some chronic cough patients had a significant increase in the percentage of eosinophils in their sputum, however, with the absence of airway hyperreactivity (AHR)^1^. The clinical features of these patients were clearly different from those of asthma patients. This disease was defined as eosinophilic bronchitis (EB). AHR presents excessive or premature contraction of the airways to various stimulating factors to induce bronchostenosis, leading to asthma patients suffering from dyspnea and wheezing. EB may manifest as an allergic disease induced by antigens, however, it lacks the typical AHR features observed in asthma. To date, EB-related clinical studies have mainly focused on epidemiology, clinical manifestations, treatment, and prognosis^2,3^. Detailed pathogenic mechanisms and evaluation of treatment strategies, especially comparing AHR with asthma, need to be investigated using animal studies. Hence, we established a relevant EB model characterized by similar clinical manifestations of EB.

Only a few EB animal models have been developed to date. Ogawa et. al. established an EB guinea pig model using the transnasal administration of polymyxin B^4^. However, due to several factors (i.e., the model did not mimic the epidemiological conditions of human EB, the feeding cost was high, and only a few biological reagents were available), their EB model was not adopted by the scientific community. Bosnjak et. al. intranasally administered different OVA concentrations (2.5 µg, 10 µg, 25 µg, and 100 µg) to construct a mouse model for EB^5^. Administration of 10 µg of OVA showed airway inflammation similar to mice with asthma but with the absence of AHR. However, no correlation studies were performed between their model and EB.

Using mice as models to study EB has several advantages. Mice share 99% of genes with humans, the relevant detection reagents are available, gene manipulation technology is mature and housing costs are lower. In addition, mice are suitable for large batch and in-depth studies. An ideal mouse EB model needs to demonstrate similar clinical features observed in EB patients. These include the following; (1) obvious coughing; (2) absence of AHR; (3) airway eosinophilic inflammation; and (4) responsiveness to steroid therapy. We previously used a low dose (10 µg) ovalbumin (OVA) intranasal administration or large particle (mass median diameter of 8.6 µm) OVA aerosol inhalation to construct mouse EB models that showed two clinical characteristics, i.e., airway eosinophilic inflammation and the absence of AHR^6,7^. Li et. al. also developed a mouse EB model with the above two clinical characteristics using a transnasal administration of polymyxin B^8^. Due to the lack of appropriate cough detection methods for mice, the effect of steroid therapy could not be assessed. Hence, the application prospect of these EB mouse models was limited. To solve this problem, we developed software to automatically detect mouse cough based on sound monitoring and respiratory airflow waveform analysis^9^.

Based on our preliminary work, mouse asthma model with AHR and EB model with airway eosinophilia but no AHR could be established by the administration of 200 µg and 10 µg OVA, respectively^6^. The present study aimed to substantiate the other two distinctive characteristics of EB: increased frequency of cough and effective steroid treatment. We selected the invasive AHR detection methodology (which required mice to be sacrificed) in the present study, hence, measurement of cough frequency was performed first.

Recent studies have demonstrated that environmental irritants and noxious stimuli could affect the airway^10^. Capsaicin has been demonstrated to significantly influence small and large airway reactions in chronic idiopathic cough and asthma patients compared to baseline, while no effect was observed in healthy controls. In these studies, impulse oscillometry and spirometry detection were immediately used after capsaicin irritation^11^. However, Satia et. al. found that capsaicin-evoked coughing had no impact on methacholine-induced bronchoconstriction in asthma patients^12^. Whether capsaicin stimulation in airway mucosa could affect AHR examination in animal models is yet to be deciphered. Hence, in the present study, we determined the appropriate time interval that was required to eliminate the disturbance of the capsaicin effect on lung resistance detection. Once the appropriate time interval was determined, we established the EB mouse model with the four clinical characteristics mentioned previously. This developed animal model will be useful to investigate the mechanism of EB pathogenesis and differentiate AHR with asthma models.

## Materials

### Experimental animals

Specific pathogen-free (SPF) grade, female, 8-week-old BALB/c mice with body weights of 22–25 g were purchased from the Experimental Animal Centre of Guangdong Province (Guangzhou, China). Mice were housed in SPF conditions in the State Key Laboratory of Respiratory Disease and had free access to OVA feed and tri-distilled water. Light–dark alternating cycles were 12/12 h.

### Critical equipment and reagents

Critical equipment and reagents included the non-invasive whole-body plethysmography system (DSI, USA), Finepointe cough detection software (version 2.2.6, jointly developed by Guangzhou State Key Laboratory of Respiratory Diseases and DSI Incorporated Company), invasive airway resistance and lung compliance detection system (RC system, DSI, USA), capsaicin (Sigma, USA), Ovalbumin (Grade V, Sigma, USA), aluminum hydroxide adjuvant (13 mg/mL, Sigma, USA), pentobarbital sodium, and methacholine (MCh, Sigma, USA), hematoxylin and eosin (HE, Zhuhai, Baso, China), and Dexamethasone (1 mL/2 mg, Guangzhou Baiyun mountain pharmaceutical co., LTD, China).

## Methods

### Part 1: Determining the optimal time interval for the detection of airway reactivity in mice after capsaicin stimulation

#### Grouping

A total of 42 mice were randomly divided into 7 groups (6 animals per group) as follows: the normal saline (NS) group, the normal saline mice with capsaicin stimulation for 6 h (NS1) group, the normal saline mice with capsaicin stimulation for 12 h (NS2) group, the asthmatic mice with capsaicin stimulation for 6 h (AS1) group, the asthmatic mice with capsaicin stimulation for 12 h (AS2) group, the EB mice with capsaicin stimulation for 6 h (EB1) group, and the EB mice with capsaicin stimulation for 12 h (EB2) group.

#### Model establishment

**Figure 1:**
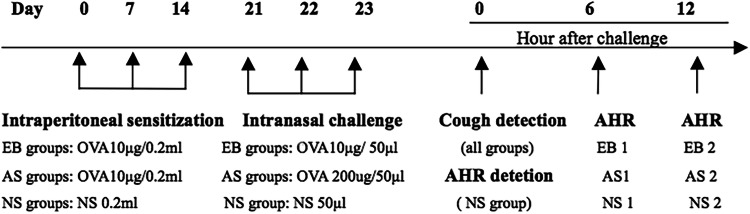
Flow chart of the study protocol for Part 1. EB groups: eosinophilic bronchitis groups, including EB1 and EB2 group. AS groups: asthma groups, including AS1 and AS2 groups. NS groups: normal saline groups, including NS, NS1, and NS2 groups.

The model was established based on our previous study^6^. The AS1, AS2, EB1, and EB2 groups were administered intraperitoneal injections of the OVA sensitization solution at 0.2 mL/animal (containing 10 µg of OVA + 0.1 mL of aluminum hydroxide adjuvant) on days 0, 7, and 14 for sensitization. On days 21, 22, and 23 (3 continuous days), each group was administered 50 mg/kg of pentobarbital sodium for intraperitoneal anesthesia, followed by intranasal stimulation. Mice in the AS1 and AS2 groups were administered 200 µg of OVA intranasal stimulation, while the EB1 and EB2 groups were administered 10 µg of OVA intranasal stimulation. Additionally, the NS, NS1, and NS2 groups were administered corresponding doses of NS for sensitization and stimulation. The flow chart of the study protocol for Part 1 is shown in Fig. 1.

#### Capsaicin stimulation and detection of airway reactivity

After establishing the mouse models for the seven groups, airway reactivity was determined for the NS group. The remaining groups were stimulated with atomized capsaicin (0.1 mmol/L) and airway reactivity was determined after 6 h and 12 h.

#### Optimal time interval for determining airway reactivity after capsaicin stimulation

This was determined based on changes in airway reactivity of all groups.

### Part 2: Establishment of an EB mouse model with the four characteristic clinical features

#### Grouping

A total of 28 mice were randomly divided into 4 groups (7 animals per group) as follows: the normal saline (NS) group, the asthma group, the eosinophilic bronchitis (EB) group, and the dexamethasone (DXM) group.

#### Model establishment

**Figure 2:**
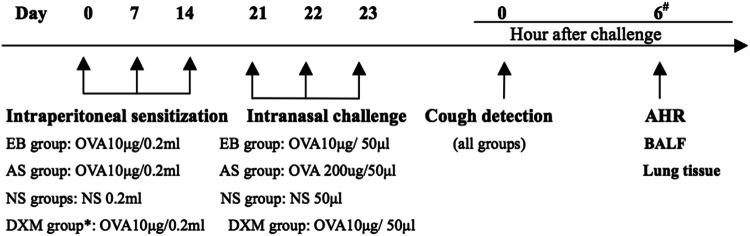
Flow chart of the study protocol for Part 2. Asterisk: DXM 5 mg/kg was administered to mice in the DXM group 1 h prior to three rounds of challenge, and AHR measurement. Hash: At 6 h post-challenge, AHR was evaluated. After evaluation, BALF and lung tissues were harvested for subsequent assessments.

The asthma, EB, and DXM groups were administered intraperitoneal injections of the OVA sensitization solution at 0.2 mL/animal (containing 10 µg of OVA + 0.1 mL of aluminum hydroxide adjuvant) for sensitization on days 0, 7, and 14. On days 21, 22, and 23, all groups were administered 50 mg/kg of pentobarbital sodium for intraperitoneal anesthesia, followed by intranasal stimulation. The asthma group received 50 µL of sensitization solution (containing 200 µg of OVA) for intranasal stimulation. Additionally, the EB and DXM groups were administered 50 µL of OVA sensitization solution (containing 10 µg of OVA) for intranasal stimulation. The DXM group was administered an intraperitoneal injection of 5 mg/kg of DXM for treatment 1 h prior to three rounds of anesthesia and intranasal stimulation and also 1 h prior to evaluation for airway reactivity. The NS group was administered 50 µL of NS intranasal stimulation. The flow chart of the study protocol for Part 2 is shown in Fig. 2.

#### Determination of cough sensitivity after model establishment

**Figure 3:**
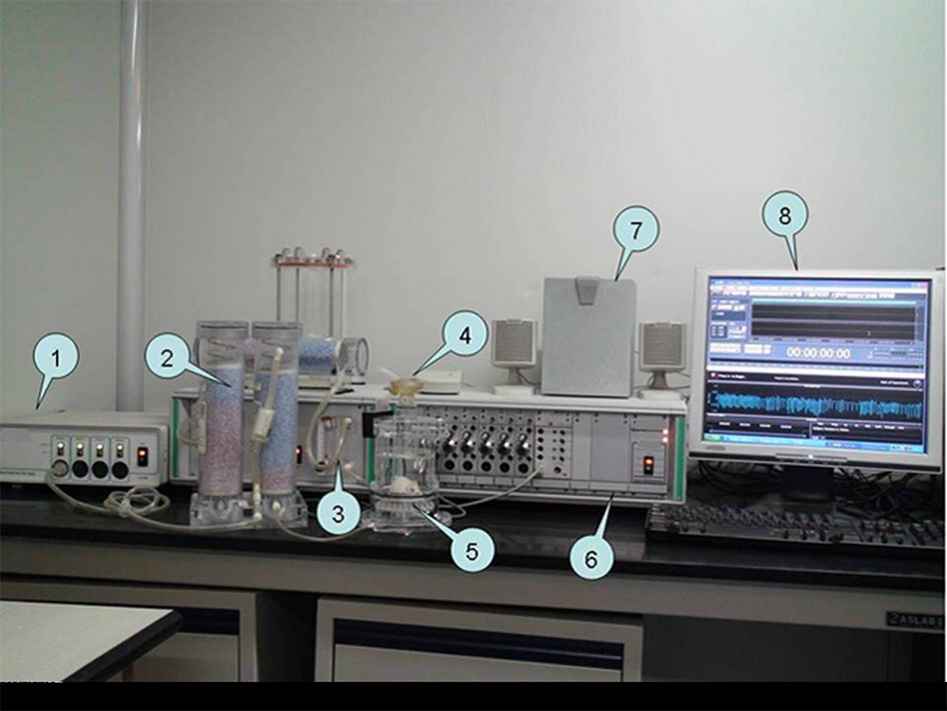
Mouse cough detection equipment. (1) Bias flow generator; (2) Desiccant; (3) Nebulizer controller; (4) Nebulizer; (5) Plethysmograph; (6) Amplifier; (7) Speakers; (8) Monitor display.

**Figure 4:**
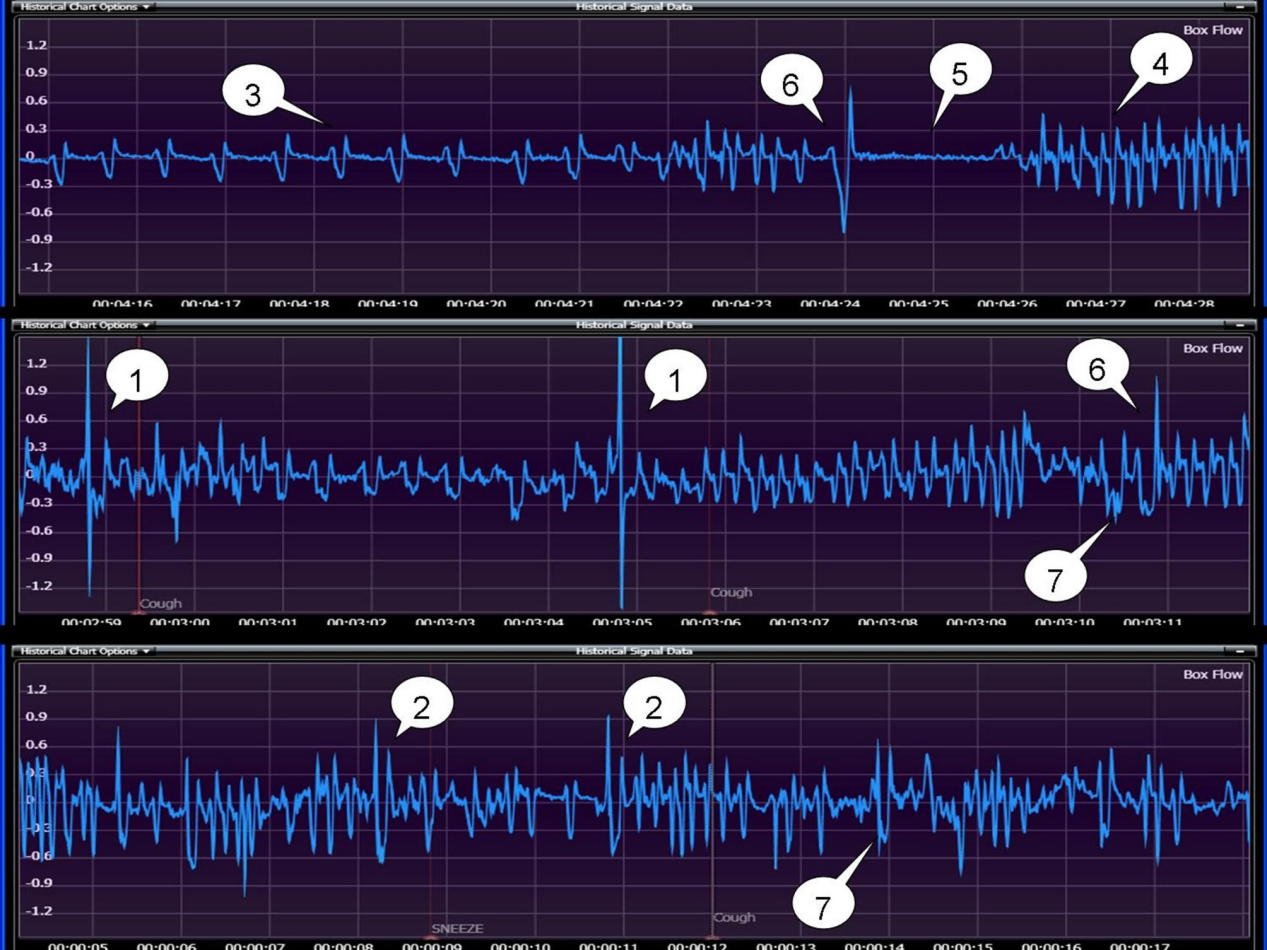
Seven types of mice respiratory waveforms: (1) cough; (2) sneeze; (3) eupnea; (4) tachypnea; (5) breath-holding; (6) deep inspiration; (7) head-twitch.

Cough sensitivity was measured in mice for all groups after model establishment and was based on the cough detection method established previously by our group. Mice were placed in a DSI non-invasive body plethysmograph chamber and then a mini-microphone was mounted onto the lateral aperture of the plethysmograph. The output port was connected to the main computer and sound waves were recorded through the Cooledit sound analysis software. Sound was analyzed through a speaker for real-time monitoring. The plethysmograph chamber was connected to a signal converter, and airflow changes in the chamber were converted to respiratory waveforms through the Finepointe cough detection software for recording and automated real-time analysis. Atomized capsaicin (0.1 mmol/L) was used for cough stimulation, and the frequency of coughs in mice for all groups prior to the model establishment was automatically detected using the Finepointe software and recorded^9^. The mouse cough detection equipment and the previous seven identified types of mouse respiratory waveforms are shown in Figs. 3 and 4.

#### Detection of airway reactivity in the mouse models

Based on the experimental results derived from Part 1, the airway reactivity of mice at 6 h post capsaicin stimulation was measured using the mouse invasive airway resistance and compliance detection system^13,14^. The procedures were as follows: mice were anesthetized using 1% pentobarbital sodium at 90 mg/kg, followed by tracheal intubation. Mice were then placed in a closed body plethysmograph chamber and a small animal ventilator was used to assist ventilation. The frequency of the ventilator was set at 120 times/min, and the tidal volume was set at 0.2 mL. Changes in lung resistance were measured after PBS stimulation and at 6.25 mg/mL, 12.5 mg/mL, and 25 mg/mL of nebulized MCh. Airway function was expressed as mean lung resistance (R_L_) = cmH_2_OmL^−1^ s^-1^ and was used to evaluate the airway reactivity of the animals.

#### Collection of bronchoalveolar lavage fluid (BALF) and cell type measurements

After measuring airway reactivity, 0.8 mL of pre-cooled PBS was injected into the lungs via tracheal intubation, and then BALF was collected by gradual withdrawal. The procedure was repeated three times. The standard recovery rate of BALF for each mouse was greater than 80%. BALF was centrifuged 3,000 r/min at 4 °C for 10 min. The precipitate was then smeared onto slides for hematoxylin and eosin (HE) staining. The total inflammatory cell score and percentages of the different cell types were calculated.

#### Lung tissue pathology

Lungs were perfused with 10% neutral formaldehyde through tracheal intubation for inflation and internal fixation. The lungs were then harvested and immersed in 10% neutral formaldehyde for external fixation. The left lung lobe was resected, treated with different concentrations of gradient ethanol and xylene, embedded in paraffin, sectioned, and stained with HE.

## Statistical analysis

Data were expressed as mean ± SD. Comparisons between groups were performed using one-way analysis of variance (ANOVA) using the SPSS 18.0 software. Multiple comparisons between groups were performed using the least significant difference (LSD) method. When the variances were not homogeneous, the rank-sum test was used. A p ˂ 0.05 denoted statistical significance.

## Results

### Part 1: Measuring airway reactivity in mice at 6 h post cough stimulation excluding the influence of capsaicin on mouse airway reactivity

**Figure 5:**
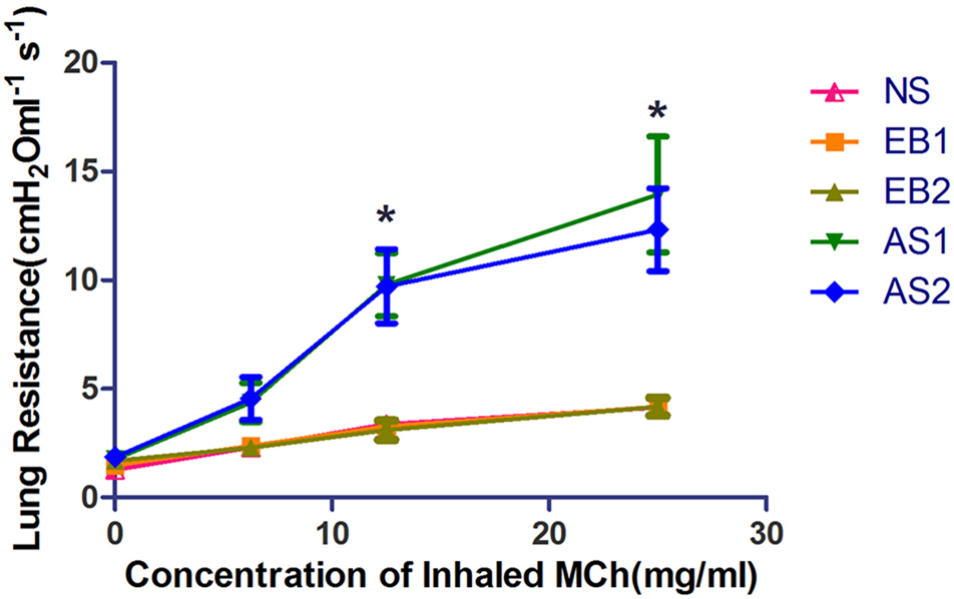
Changes of lung resistance in the asthma and EB groups after capsaicin stimulation at 6 h and 12 h. Lung resistance in the EB1 and EB2 groups after MCh nebulization (6.25, 12.5, and 25 mg/mL) were not significantly different compared to the NS group and did not significantly differ between the two groups. After MCh (6.25–25 mg/mL) nebulization, lung resistance was significantly lower compared to the AS1 and AS2 groups (*p < 0.01; LSD method). Lung resistance did not significantly differ between the AS1 and AS2 groups at all MCh concentrations. Data were expressed as mean ± standard deviation (six animals per group).

**Figure 6:**
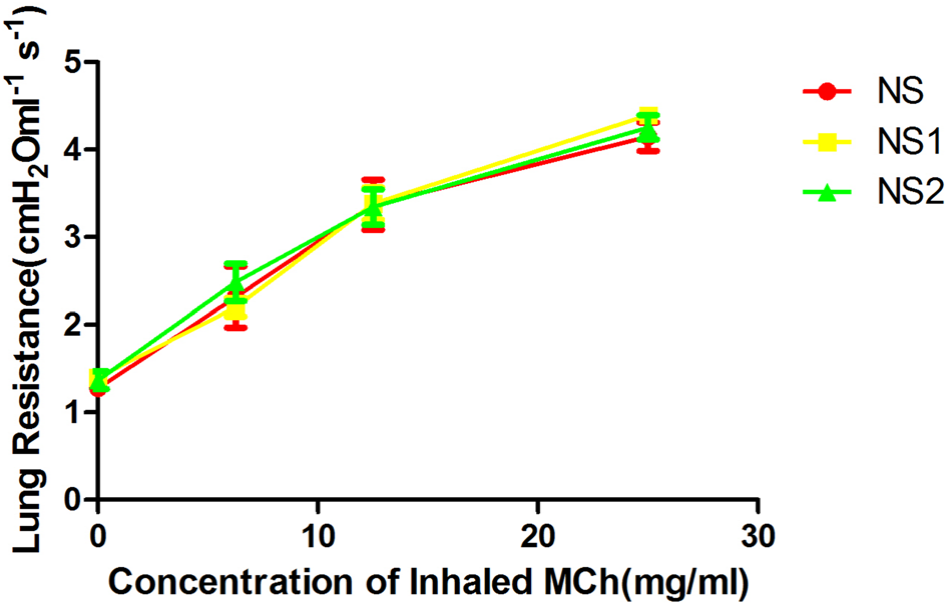
Changes in lung resistance in the NS1, NS2, and NS groups after capsaicin stimulation at 6 h and 12 h. After MCh (6.25–25 mg/mL) stimulation, lung resistance in the NS1 and NS2 groups did not significantly differ compared to the NS group. Data were expressed as mean ± standard deviation (six animals per group).

The lung resistance in the EB1 and EB2 groups after MCh nebulization (6.25, 12.5, and 25 mg/mL) was not significantly different compared to the NS group (p > 0.05). In addition, lung resistance was not significantly different between these two groups (p > 0.05). After MCh nebulization (12.5 and 25 mg/mL), lung resistance was significantly higher in the AS1 and AS2 groups compared to the NS, EB1, and EB2 groups (p < 0.01). However, lung resistance did not significantly differ between the AS1 and AS2 groups (p > 0.05) (Fig. 5). Lung resistance in the NS1 and NS2 groups was not significantly different after MCh (6.25–25 mg/mL) stimulation compared to the NS group (p > 0.05) (Fig. 6).

### Part 2: Cough frequency is significantly increased in the asthma and EB groups, while cough sensitivity is significantly decreased after steroid therapy

**Figure 7:**
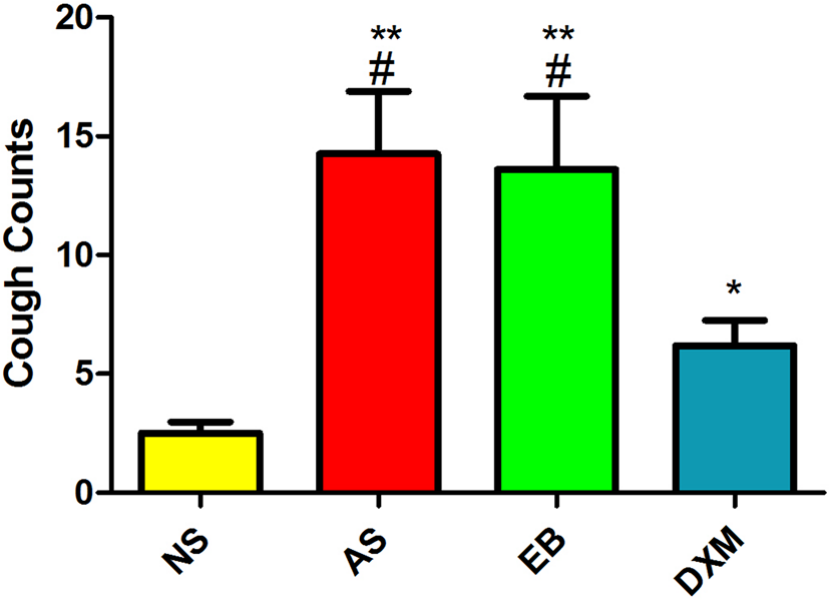
Changes in cough sensitivity in mice for the four groups after model establishment. The frequency of coughs in the asthma and EB groups were significantly higher compared to the NS group (^#^p < 0.01; ANOVA) and were significantly higher compared to the DXM group (**p < 0.05; ANOVA). The frequency of coughs in the DXM group was significantly higher compared to the NS group (*p < 0.05; ANOVA). Data expressed as mean ± standard deviation (seven animals per group).

After model establishment, the frequency of coughs in the asthma group (14.25 ± 9.13 times) and the EB group (13.60 ± 9.75 times) was significantly higher compared to the NS group (2.10 ± 1.10 times) (p < 0.01). The frequency of coughs in the DXM group (6.2 ± 3.36 times) was significantly reduced compared to the asthma and EB groups (p < 0.05) (Fig. 7).

### AHR does not manifest in the EB group

**Figure 8:**
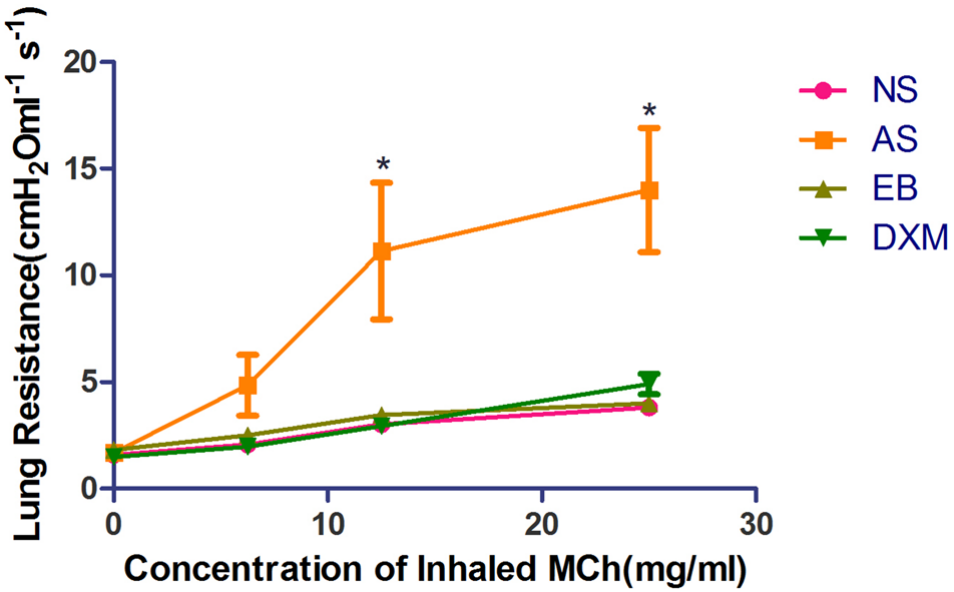
Airway reactivity in mice from the four groups after model establishment. After MCh (12.5–25 mg/mL) administration, lung resistance in the asthma group was significantly higher compared to the other three groups (*p < 0.05; LSD method). Data expressed as mean ± standard deviation (seven animals per group).

Lung resistance in the asthma group after MCh nebulization (12.5 and 25 mg/mL) was significantly higher compared to the other three groups (p < 0.05). The differences in lung resistance among the EB, NS, and DXM groups after MCh (6.25–25 mg/mL) stimulation were not significant (p > 0.05) (Fig. 8).

### Total number of inflammatory cells and EOS percentages were significantly higher in the BALF of mice in the asthma and EB groups but were significantly reduced after steroid therapy

**Figure 9:**
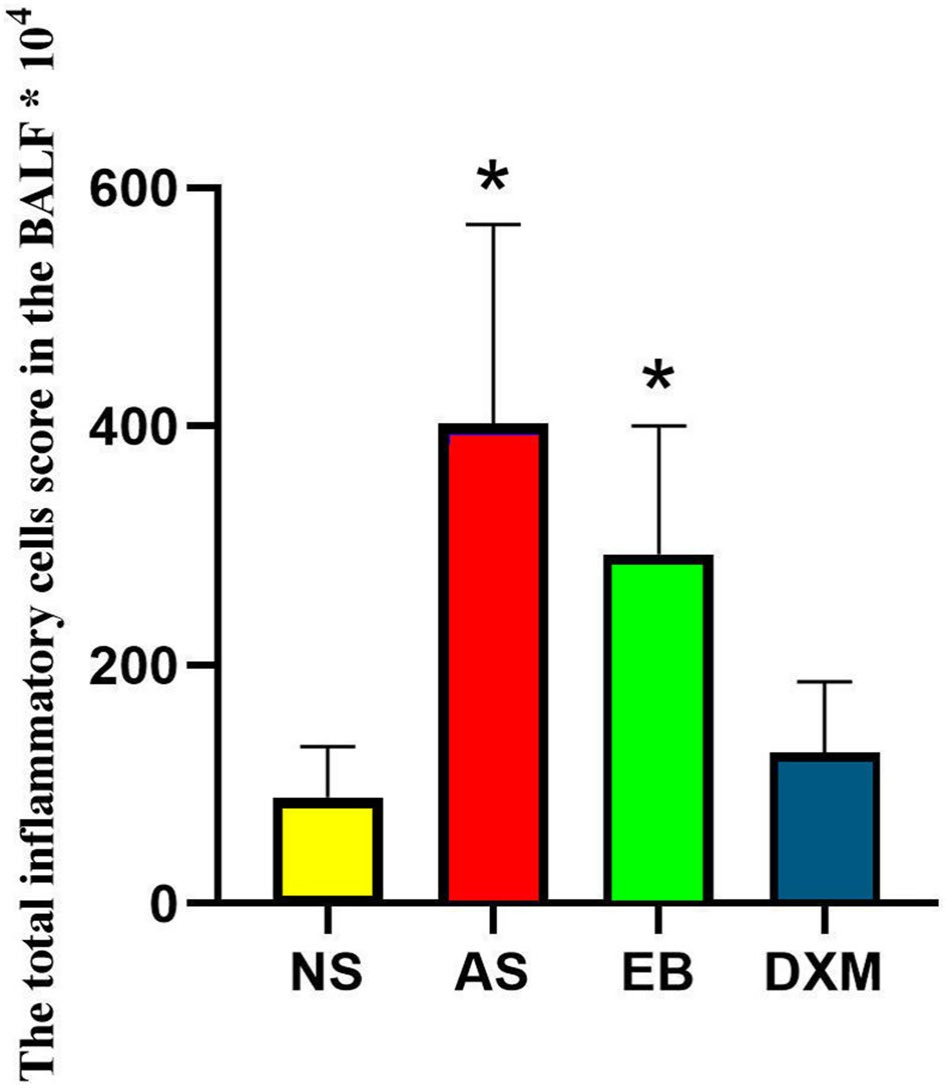
The total inflammatory cell score in the BALF of mice for the four groups. The total inflammatory cell score in the BLAF of mice from the asthma and EB groups were significantly higher compared to the NS and DXM groups (*p < 0.05; ANOVA). Data expressed as mean ± standard deviation (seven animals per group).

**Figure 10:**
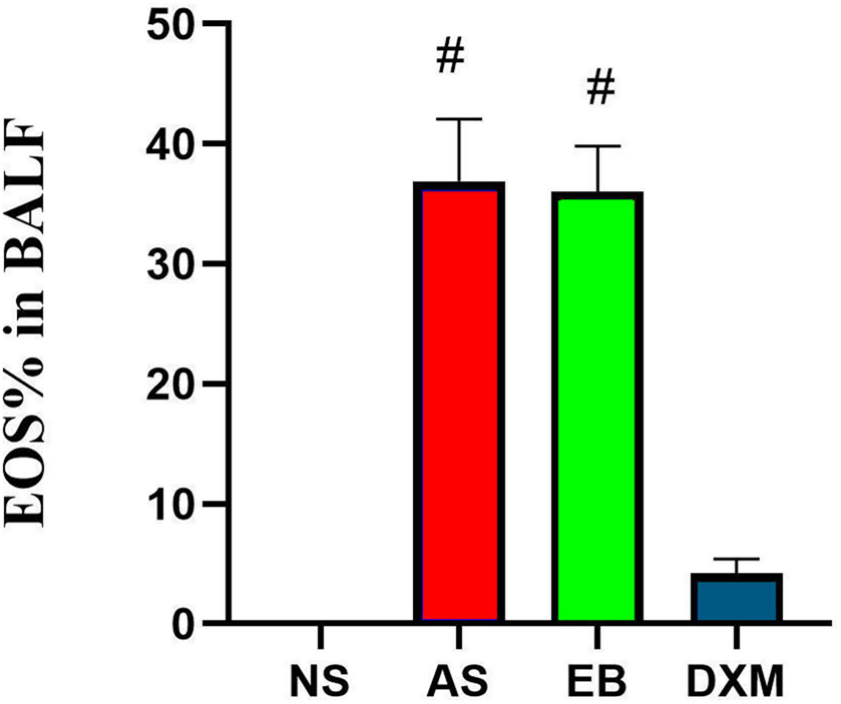
EOS percentage in the BALF of mice from the four groups. The percent of EOS in the BALF of mice from the asthma and EB groups was significantly higher compared to the NS and DXM groups (^#^p < 0.01; ANOVA). Data expressed as mean ± standard deviation (seven animals per group).

**Figure 11:**
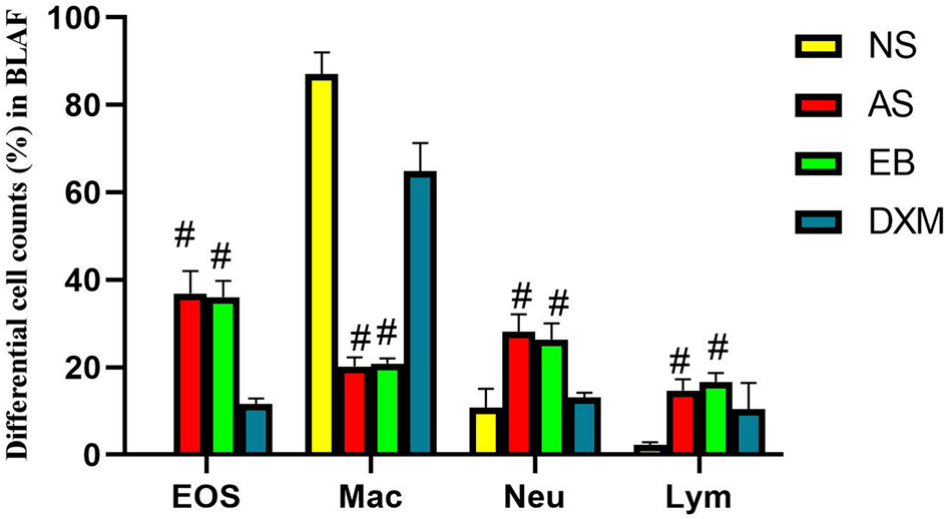
Differential cell counts in BALF. Eosinophil, neutrophil, and lymphocyte counts were increased significantly in the asthma and EB groups compared to the NS group, while the macrophage counts were reduced. DXM administration decreased these changes (^#^p < 0.01; ANOVA). Data expressed as mean ± standard deviation (seven animals per group).

The total inflammatory cell score in the BALF of mice in the asthma (402.3 × 10^4^ ± 167.2 × 10^4^) and EB (292.5 × 10^4^ ± 107.6 × 10^4^) groups were higher compared to mice in the NS (89.1 × 10^4^ ± 42.8 × 10^4^) and DXM (126.9 × 10^4^ ± 59.3 × 10^4^) groups (Fig. 9). The EOS percentages in the BALF of mice in the asthma (36.89 ± 5.17%) and EB groups (36.04 ± 3.78%) after model establishment were significantly higher compared to mice in the NS (0%) and DXM groups (4.23 ± 1.16%) (p < 0.01) (Fig. 10). OVA administration increased eosinophil, neutrophil, and lymphocyte counts in the BALF of mice in the EB and asthma groups compared to the NS group, while DXM administration decreased these changes (Fig. 11).

### Pathological changes in lung tissues

**Figure 12:**
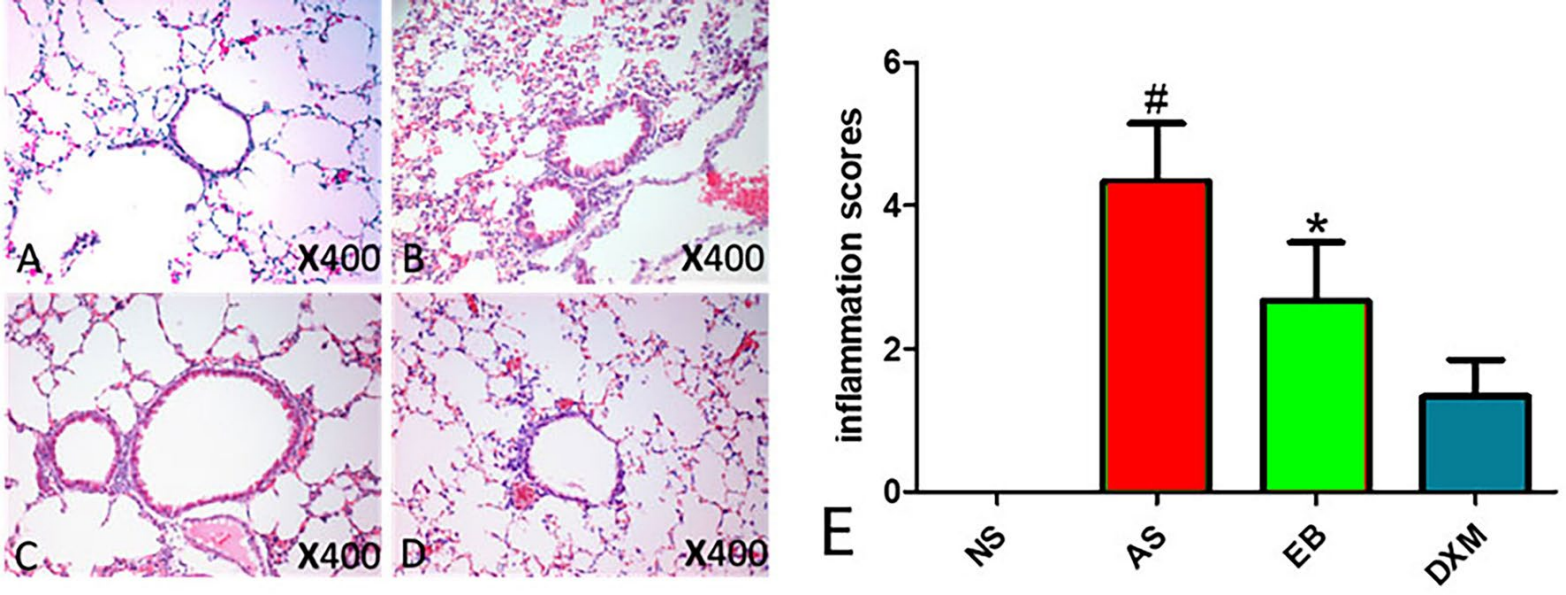
Obvious airway inflammation observed in the asthma and EB groups. After steroid therapy, airway inflammation was significantly reduced in the EB group. **A** Pathological changes in the lung tissues of mice in the NS group. **B** Pathological changes in the lung tissues of mice in the asthma group. **C** Pathological changes in the lung tissues of mice in the EB group. **D** Pathological changes in the lung tissues of mice in the DXM group. **E** The degree of inflammation around the airway expressed as inflammation scores. Inflammatory scores in the AS group were significantly higher compared to the other three groups (^#^p < 0.05). The degree of inflammation around the airway in the EB group was lower compared to the AS group, and was significantly higher compared to the DXM and NS group (*p < 0.05).

Obvious EOS infiltration in the lung tissues was observed in mice from the asthma and EB groups compared to mice in the NS group. After DXM administration, EOS infiltration in the lung tissues and inflammatory scores were significantly reduced (Fig. 12).

## Discussion

There are well-established mouse models for asthma using OVA sensitization and have been used in a large number of studies^15–17^. Epidemiological studies have demonstrated that females are more likely to develop asthma and are more hyperresponsiveness compared to males^18^. Mice asthma models have also demonstrated that Th2 cytokine production is increased to a greater extent in female mice compared to males after OVA inhalation^19^. Another advantage of using female mice to establish mouse models include them being more docile compared to male mice. This makes it easier to generate mouse models, and hence, female mice were selected for our studies.

Although EB is very similar in symptoms of asthma, the main difference is the absence of AHR. To-date, reliable EB animal models are lacking, which accordingly, have resulted in a very limited number of animal studies being performed. Asthma is a chronic disease, however, the majority of studies have been performed based on acute asthma models for AHR and airway inflammation. Our present EB model mimics the acute asthma model establishment process and aimed to further investigate the AHR mechanism and its correlation with airway inflammation. Humans sense discomfort and express their symptoms subjectively. However, for objective and rapid evaluation, cough reactive sensitivity is often used in scientific studies, with C2 and C5 preferred to indicate cough sensitivity^10^. Coughs are too weak to be heard from mice, hence, methods to detect cough should be measured objectively via sensitive instruments. Guinea pig models have been used widely in cough studies, with capsaicin or other stimuli often used to induce coughs^20,21^. In this study, capsaicin was selected to induce mouse coughs.

To further develop the mouse EB model, we successfully created the mouse cough detection software as a prerequisite^9^. At present, no studies have investigated whether capsaicin to induce mouse cough sensitivity influences airway reactivity. To ensure a successful establishment of the asthma mouse model with AHR and the EB model without AHR, the first part of this study was to evaluate changes in airway reactivity in mice after detection of cough induced by capsaicin after 6 and 12 h. This was performed to select the optional time point to determine airway reactivity after capsaicin stimulation. Results showed that the airway reactivity did not significantly differ in mice from the NS, NS1, and NS2 groups. This indicated that capsaicin stimulation did not influence airway reactivity in mice in the normal control group, and was similar to capsaicin effects in healthy individuals^11^. However, whether capsaicin stimulation affected the airway reactivity in mice in the EB or asthma group, which had airway inflammation, was unknown. In addition, what appropriate time interval was required to eliminate the effects of cough on airway reactivity was unknown. There were no significant differences in the EB1 and EB2 groups compared to the NS groups at any time point. This suggested the successful generation of the EB mouse model without AHR. Additionally, airway reactivity did not significantly differ between these two groups at 6 and 12 h after capsaicin stimulation. This suggested that the time to detection of airway reactivity in EB mice, at least after 6 h of cough stimulation, could exclude the influence of capsaicin on airway reactivity. Similarly, the influence of capsaicin stimulation on airway reactivity was measured in asthmatic mice. Our results demonstrated that airway reactivity after capsaicin stimulation for 6 and 12 h in the AS1 and AS2 groups, with 12.5 mg/mL and 25 mg/mL of MCh, were significantly higher compared to the NS group. This indicated that the asthma model with AHR was established successfully. Airway reactivity did not significantly differ between the AS1 and AS2 groups, suggesting that capsaicin did not interfere with a successful model establishment in asthmatic mice. Additionally, both 6 h and 12 h were optional time points for the measurement of airway reactivity. Johansson et. al. demonstrated that capsaicin-induced chronic idiopathic cough and lung resistance in asthma patients, especially on the small airways^11^. In their study, lung resistance was evaluated right after capsaicin stimulation. While in our study, there were no effects of capsaicin on any of the model groups and was consistent with the study performed by Satia et al.^12^. This may be due to the following three reasons; first, our study was performed at a time interval of 6 and 12 h, and was not performed immediately between cough detection and airway reactivity; second, the detection method was within the large airway, with certain small changes not being easily detected; third, the acute mouse EB and asthma models were developed in this study and not chronic models, which may have contributed to the differences. Since we aimed to eliminate the effect of capsaicin on model establishment, 6 h was selected as the time interval between the two measurements for Part 2 of the study.

Part 2 involved the comprehensive examination of whether mice in the EB group had all of the clinical features of EB (increased cough, airway inflammatory cell infiltration, absence of AHR, and responsiveness to steroid therapy). After model establishment, the frequency of coughs in the EB group was significantly higher compared to the NS group. The frequency of coughs in the DXM group was significantly reduced compared to the EB group. These results suggested that cough sensitivity in the EB group was significantly increased compared to the NS group, and DXM administration could reduce the cough sensitivity in the EB mice. However, the frequency of coughs in the DXM group was still higher compared to the NS group. This may be related to suboptimal steroid administration in the acute model. After stimulation using concentrations of 12.5 mg/mL and 25 mg/mL of MCh, airway reactivity of the mice in the asthma group were significantly increased compared to the other three groups. However, no significant differences in airway reactivity were observed between the EB and NS groups. These results suggested that the asthma group but not the EB group developed AHR. Total inflammatory cell scores and EOS percentages in the BALFs of mice from the asthma and EB groups were significantly higher compared to the DXM and NS groups. Pathological assessment of the lung tissues demonstrated that the asthma and EB groups had obvious eosinophil infiltration in the small airways and lung tissues, whereas airway inflammation in the DXM group was significantly reduced. Hence, we demonstrated that mice in the EB group had all of the desired clinical features of EB, including the increased frequency of coughs, airway inflammatory cell infiltration, absence of AHR, and responsiveness to steroid therapy.

Asthma and EB are both common aetiologies of chronic cough^22–24^. Several studies have demonstrated that development of chronic cough in asthma patients may be associated with airway eosinophilic inflammation, epithelial damage, airway remodeling, and goblet cell hyperplasia^25–27^. Studies on the pathogenic mechanism of chronic cough in EB are relatively rare and have been mainly focused on clinical studies^28–30^. Brightling et al. showed that cough sensitivity was significantly improved in clinical EB patients who received inhaled steroid therapy for 4 weeks at the time of reduction of airway eosinophilic inflammation^28^. Ma et. al. observed the effect of inhaled steroid therapy for 4 weeks in 86 chronic cough patients diagnosed with EB^29^. They found that airway eosinophilic inflammation was significantly improved, and cough symptoms disappeared during the final week of steroid therapy. Hence, similar to asthma patients, chronic cough in EB patients may be associated with airway eosinophilic inflammation. Our results demonstrated that the frequency of coughs in the DXM group was significantly lower compared to the EB group and airway eosinophilic inflammation in the DXM group was significantly improved compared to the EB group. These results suggested that steroid therapy not only attenuated airway eosinophilic inflammation in EB mice but also improved cough sensitivity. This is consistent with previously published studies. To date, no animal studies have been performed to investigate whether AHR and cough influence each other. Our study demonstrated that the frequency of coughs did not significantly differ between mice in the EB and asthma groups. These results suggested that the degree of cough may not correlate with AHR, but may be associated with airway eosinophilic infiltration.

The most common symptoms in typical asthmatic patients are wheezing and paroxysmal dyspnea. Cough variant asthma (CVA) is recognized as a precursor of typical asthma^31^. However, cough presentation differs between these similar diseases. Chen et. al observed differences in small airway function between CVA and typical asthma patients^32^. Yamamura et al. found that repeated bronchoconstriction leads to decreased cough reactions in guinea pig asthma models and endogenous lipid mediators contribute to the difference between CVA and typical asthma^33^. Only a few studies have investigated the degree of cough differences between EB and CVA patients. At present, cough counters used in clinical practice lack objectivity and are unable to accurately determine cough numbers and the degree of cough severity. In this study, both EB and asthma mouse models had increased frequency of coughs compared to the NS group, suggesting the generation of a successful CVA mouse model. Increased eosinophilia infiltration and frequency of cough were similar between the EB and asthma groups. DXM administration (DXM group) reduced eosinophilia infiltration and frequency of cough, suggesting that eosinophilia maybe be associated with cough sensitivity. Additional studies are required to better understand the underlying mechanisms. The differences in cough sensitivity and airway reactivity among EB, CVA, and typical asthma needs further study. Our mouse EB model will help answer these differences.

In summary, we were the first to establish an EB mouse model that showed the clinical manifestations of cough, absence of AHR, with airway eosinophilic inflammation, and was responsive to steroid therapy. The model was similar to those clinical features observed in EB patients. This model will be very helpful to investigate the mechanism, outcomes, treatment strategies, and new drug development for EB. In addition, the model will be useful to understand AHR mechanism as it relates to airway responsiveness differences in asthma.

### Ethics approval

The study conforms with the Guide for the Care and Use of Laboratory Animals published by the US National Institutes of Health (NIH Publication No. 85-23, revised 1996). The study protocol was approved by the Animal Ethics Committee of Guangzhou Medical University.

## Data Availability

The datasets used and/or analyzed in the current study are available from the corresponding author on reasonable request.
